# Rare diseases caused by abnormal calcium sensing and signalling

**DOI:** 10.1007/s12020-021-02620-5

**Published:** 2021-02-02

**Authors:** Judit Tőke, Gábor Czirják, Péter Enyedi, Miklós Tóth

**Affiliations:** 1 Department of Internal Medicine and Oncology, Semmelweis University, Budapest, Hungary; 2 Department of Physiology, Semmelweis University, Budapest, Hungary

**Keywords:** Calcium-sensing receptor, Autosomal dominant hypocalcemia, Familial hypocalciuric hypercalcemia, Neonatal severe hyperparathyroidism

## Abstract

The calcium-sensing receptor (CaSR) provides the major mechanism for the detection of extracellular calcium concentration in several cell types, via the induction of G-protein-coupled signalling. Accordingly, CaSR plays a pivotal role in calcium homeostasis, and the *CaSR* gene defects are related to diseases characterized by serum calcium level changes. Activating mutations of the *CaSR* gene cause enhanced sensitivity to extracellular calcium concentration resulting in autosomal dominant hypocalcemia or Bartter-syndrome type V. Inactivating *CaSR* gene mutations lead to resistance to extracellular calcium. In these cases, familial hypocalciuric hypercalcaemia (FHH1) or neonatal severe hyperparathyroidism (NSHPT) can develop. FHH2 and FHH3 are associated with mutations of genes of partner proteins of calcium signal transduction. The common polymorphisms of the CaSR gene have been reported not to affect the calcium homeostasis itself; however, they may be associated with the increased risk of malignancies.

## Physiological role of calcium-sensing receptor (CaSR) and its partner signalling proteins

The CaSR is a member of family C of G-protein-coupled receptors. It was first cloned from bovine parathyroid cells in 1993 [1]. The human *CaSR* gene localizes on chromosome 3q13.3-21 and contains 8 exons, from which the first (1A and 1B) encodes alternatively spliced 5′-untranslated regions. The receptor protein is constituted of 1078 amino acids. It contains a large extracellular domain responsible for ligand binding and receptor dimerization, the transmembrane domain comprising seven transmembrane helices and a smaller intracellular domain, which transduces the evoked signal to the downstream intracellular partner proteins [2]. The receptor is highly expressed on the chief cells of the parathyroid glands and in the kidney; however, its expression has also been demonstrated in numerous other tissues and cell types [3].

The investigation of wild-type and mutated CaSR activity, complemented with the data of recent structural analyses of CaSR, provided deeper insight into the process of calcium-sensing and signalling. The activation of the dimerized receptor upon ligand binding causes conformational changes and coupling to Gα_q/11_ proteins. The main intracellular effector of this signal transduction is the cytoplasmic ionized calcium [4]. Proper expression on the cell surface is required for the normal function of the CaSR. The level of cell-surface expression is influenced by the balance of agonist-driven insertional signalling (ADIS) and the activity of adaptor-related protein complex 2 (AP2). ADIS provides anterograde trafficking of newly synthesised receptors to the plasma membrane after prolonged receptor activation. AP2 together with β-arrestin represent the key determinants of elimination from the cell surface as they facilitate clathrin-mediated endocytosis of CaSR [5].

## Inherited calcitropic diseases of the CaSR and its partner proteins

The disturbances of extracellular calcium-sensing lead to pathological hypersensitivity or resistance to the extracellular calcium resulting in several disease states. The majority of these disorders is genetically determined by the mutations of the *CaSR* and its downstream partner signalling proteins. Some of them activate the CaSR resulting in hypersensitivity to extracellular calcium. This group of inherited hypocalcemic disorders involves the autosomal dominant hypocalcemia (ADH) (ADH1 and ADH2) and Bartter-syndrome type V. Other genetic defects inactivate the calcium sensing pathway leading to resistance to extracellular ligands of CaSR. Heterozygous loss-of-function mutations lead to one of the three known subtypes of the usually symptomless familial hypocalciuric hypercalcaemia (FHH), while germ-line homozygous inactivating *CaSR* gene mutations are in the background of neonatal severe hyperparathyroidism (NSHPT).

### Diseases associated with increased sensitivity to the extracellular calcium concentration

#### Autosomal dominant hypocalcemia (ADH)

Diseases with increased sensitivity to extracellular calcium are characterized by low extracellular calcium concentrations with inappropriately normal or suppressed parathyroid hormone (PTH) secretion. The prototype of these conditions is the ADH representing the most common genetic cause of isolated hypoparathyroidism.

ADH1 (OMIM: 601198) accounts for ≈70% of ADH cases and are caused by heterozygous gain-of-function *CaSR* gene mutations [6]. It is considered as a rare disorder with prevalence 3.9 per 100,000 [7]. In the affected patients, the main laboratory findings are the subnormal or normal serum PTH level, together with inappropriately normal or elevated urinary calcium excretion despite low serum calcium. About half of the patients have mild hypocalcemic symptoms (neuromuscular irritability), and ectopic calcification (including basal ganglia) may also develop in about 35% of patients [8]. Thiazide diuretics, calcium- and activated vitamin D supplementation can be offered to symptomatic individuals. The goal of the treatment is to maintain serum calcium level slightly below the normal range in order to avoid marked hypercalciuria, nephrocalcinosis, nephrolithiasis and renal impairment [9]. Recent studies aim to evaluate the safety and efficacy of calcilytic drugs in patients with ADH1. To date, there are clinical data with intravenously administered NPSP795, a negative allosteric modulator of the CaSR. Observations obtained from five patients with ADH1 confirmed the initial expectations that the short-acting NPSP795 can significantly and rapidly increase PTH secretion in a dose-dependent manner [10].

ADH2 (OMIM: 615361) is caused by activating heterozygous *GNA11* gene mutations. *GNA11* gene, located on chromosome 19p13.3, encodes the G-protein subunit α_11_ (Gα_11_) protein, a major mediator of CaSR signalling. Eight heterozygous germ-line mutations of *GNA11* gene have been reported to date. Patients with ADH2 have a milder biochemical phenotype than those with ADH1 [11]. In two kindreds, short stature was also observed [12, 13].

#### Bartter-syndrome type V

Bartter syndrome is characterized by deficient renal reabsorption of sodium and chloride, as well as hypokalemic metabolic alkalosis with secondary hyperaldosteronism. Genetic alterations of several ion transporters and channels have been associated with the pathogenesis of Bartter’s syndrome, including activating mutations of the CaSR [14]. In Bartter-syndrome type V (OMIM: 601198), the constitutive activation of CaSR in the thick ascending limb inhibits the activity of the renal outer-medullary K channel and this results in renal salt wasting, hypocalcemia and hypomagnesemia as well as hyperreninaemic hyperaldosteronism and hypokalaemic alkalosis [15].

### Diseases associated with decreased sensitivity to the extracellular calcium concentration

#### Familial hypocalciuric hypercalcemia (FHH)

Inactivating mutations in the proteins of calcium-sensing and signalling pathway cause FHH. FHH is a genetically heterozygous group of disorders inherited by autosomal dominant trait and characterized by mild, usually symptomless hypercalcemia and relative hypocalciuria [16]. At present, three types of FHH are known (FHH1, FHH2, FHH3). Their relative prevalances were estimated as 64:1:10, respectively [17].

FHH1 (OMIM: 145980) is caused by germ-line loss-of-function *CaSR* gene mutations on chromosome 3q21.1. This is the most common form of FHH comprising about 85% of all FHH cases. Over 300 *CaSR* gene mutations have been reported to date. Most of them are missense substitutions. The genetic defect usually affects the first 350 amino acid residues of the extracellular domain of the receptor. The altered structure of the extracellular domain is responsible for the decreased affinity to ionized calcium and impaired receptor dimerization. Previous studies have revealed that 50% of *CaSR* gene mutations affect receptor trafficking and cell-surface expression [18]. Clinically, FHH1 is characterized by mild, non-progressive hypercalcemia, normal (in 80% of cases) or slightly elevated (in 20% of cases) serum PTH levels and hypocalciuria featured by low (<0.01) calcium-to-creatinine clearance ratio (CaCrCR) [19]. The majority of patients are symptomless, though some with moderate-to-severe hypercalcemia may present with classical hypercalcemic symptoms (polyuria-polydipsia, fatigue, chronic pancreatitis, gallstones and chondrocalcinosis). In these latter rare cases, the affected patients may benefit from calcimimetic drug treatment [20, 21]. FHH1 have been widely considered as a rare condition; however, according to a recent publication, the estimated frequency of FHH1 would be as high as the age-adjusted prevalence of classical primary hyperparathyroidism (PHPT): 74.1/100,000 for FHH1 vs. 48/100,000 for PHPT in adult males and 120/100,000 for PHPT in adult females [7, 22]. These findings strengthen previous recommendations, that in clinical practice, FHH1 should be differentiated from mild forms of PHPT as parathyroidectomy indicated for PHPT will not improve hypercalcemia in FHH patients. The first step to differentiate PHPT and FHH is the determination of CaCrCR. However, diagnosing patients with CaCrCR in the grey zone (0.01–0.02) is still challenging and needs genetic investigations [23].

Occasionally, neonates with heterozygous de novo or paternally inherited *CaSR* gene mutation could present with severe clinical symptoms resembling to NSHPT (described below in detail). In these cases, the affected fetuses perceive the normal maternal calcium as low; therefore, secondary hyperparathyroidism (SHPT) will develop during intrauterine life [24]. Following delivery, as the maternal trigger to excess secretion of PTH is no longer present, the neonate will increase his/her calcium concentration to a higher value within a few days–weeks, and the rate of the PTH secretion will return to normal [25]. As a consequence, despite the severe laboratory signs and clinical symptoms resembling to NSHPT, this special, self-limiting form of hypocalciuric hypercalcemia should be classified as FHH1.

Genetic linkage analyses revealed that loss-of-function gene mutations of proteins involved in downstream CaSR-signalling pathway cause FHH2 and FHH3.

FHH2 (OMIM: 145981) is a rare autosomal dominant disorder caused by germ-line inactivating mutations of the *GNA11* gene, encoding the G_α11_ protein. To date, three different *GNA11* gene mutations have been reported (Thr54Met, Leu135Gln, Ile200del). All patients presented with mild, symptomless hypercalcemia (<2.80 mmol/L) [6, 26]. According to a recent study, the calcimimetic drug cinacalcet may have the potential to correct the hypercalcemia caused by the inactivating *GNA11* Phe220Ser gene mutation, perhaps by enhancing the signalling process through the wild-type copy of G_α11_ protein [27].

In FHH3 (OMIM: 600740), germ-line mutations of *AP2S1* gene located on chromosome 19q13.3 are present. *AP2S1* gene encodes the sigma subunit of the adaptor-related protein-2 (AP2σ), which has a fundamental role in clathrin-mediated CaSR endocytosis. The mutant AP2σ will lead to impaired G_α11_–mediated CaSR signalling despite increased CaSR cell-surface expression [28, 29]. To resolve this paradox, a recent study has confirmed the previous hypothesis that CaSR does not only evoke an immediate response from the plasma membrane after extracellular ligand binding, but also has a sustained signalling from the endosomes, similarly to other GPCRs. Therefore, decreased endocytosis of the CaSR from the cell surface may have a consequent negative effect on endosome-mediated signalling [30]. Interestingly, all the reported *AP2S1* gene mutations affect the R15 residue (Arg15Cys, Arg15His and Arg15Leu). Clinically, FHH3 patients usually have more severe hypercalcemia, hypermagnesemia as well as more marked hypocalciuria compared to those with FHH1. Furthermore, previous analyses revealed a genotype–phenotype correlation in the affected patients as those harbouring the Arg15Leu mutation are presented with the most severe hypercalcaemia among FHH3 patients. Low bone mineral density and cognitive disorders have also been reported in FHH3 [31].

#### Neonatal severe hyperparathyroidism (NSHPT)

NSHPT (OMIM: 239200) is caused by homozygous or compound heterozygous inactivating mutations of the *CaSR* gene. The clinical manifestation may be life-threatening severe hypercalcemia (>3.5 mmol/L) with high serum PTH level, respiratory distress, hypotonia and severe hyperparathyroid bone disease with spontaneous bone fractures could develop in the affected neonates [32, 33]. Parathyroidectomy is a reasonable therapeutic option. Medical treatment with intravenous pamidronate was reported as safe and effective rescue therapy for the management of preoperative severe hypercalcemia and life-threatening bone demineralisation [34, 35]. Cinacalcet, allosteric modulator of the CaSR also provides an alternative and safe treatment option as rapid normalization of elevated serum calcium and PTH level was reported in several cases [36–38]. There are two possible scenarios in which NSHPT could occur. (1) In neonates with homozygous CaSR mutation who are offspring of consanguineous parents harbouring the same inactivating CaSR mutation. (2) In neonates of parents, harbouring different inactivating mutations, compound heterozygous CaSR mutation could occur.

Mutations of the CaSR and its partner proteins, as well as the associated disorders, are presented in Table 1.

**Table 1:** Mutations of calcium-sensing receptor, its partner proteins and associated disorders

Protein	Gene	Chromosomal location	Disorder	OMIM	Inheritance
CaSR	*CaSR*	3q21.1			
	Inactivating mutations		FHH1	145980	Autosomal dominant
			NSHPT	239200	Autosomal recessive or dominant
	Activating mutations		ADH1	601198	Autosomal dominant
			Bartter-syndrome type V	601198	Autosomal dominant
G-protein subunit α11	*GNA11*	19p13.3			
	Inactivating mutations		FHH2	145981	autosomal dominant
	Activating mutations		ADH2	615361	autosomal dominant
AP2σ protein	*AP2S1*	19q13.3			
	Inactivating mutations		FHH3	600740	autosomal dominant

*CaSR* calcium-sensing receptor, *FHH* familial hypocalciuric hypercalcemia, *NSHPT* neonatal severe hyperparathyroidism, *ADH* autosomal dominant hypocalcemia, *AP2σ* adaptor-related protein complex 2, sigma 1 subunit

## Genetic counselling

Genetic counselling and germ-line testing of the *CaSR* gene are most frequently offered to adult patients diagnosed with asymptomatic mild hypercalcemia and hypocalciuria in whom there is a high suspicion of a hereditary disorder (familial aggregation of asymptomatic hypercalcemia, young age at the diagnosis) [39]. In clinical practice, the differential diagnosis between PHPT and FHH1 may be difficult. There are many overlaps between these two clinical entities, as PHPT could be present in an asymptomatic, mild form and hypocalciuria could be detected in some patients. On the contrary, 20% of patients with FHH1 have elevated serum PTH levels and a CaCrCR > 0.01. The proper differential diagnosis between FHH and PHPT is essential, as parathyroidectomy will not cease the hypercalcemia of FHH patients. Therefore, CaSR mutational screening would be useful in FHH1-suspected cases with atypical clinical presentation to avoid unnecessary parathyroidectomy.

The clinical presentation in FHH2 is usually similar to that of FHH1. Targeted genetic testing may be recommended for those patients in whom mutational analysis of *CaSR* gene detected normal sequences.

Concerning FHH3, mutation analysis of AP2S1 gene should be performed in those FHH patients who have marked hypermagnesemia, cognitive impairment and low bone mineral density.

In cases of the severe, life-threatening NSHPT occurring at birth or within the first 6 months of life, the diagnosis is usually established by the life-threatening clinical manifestations (severe hypercalcaemia, hypotonia, bone demineralization, fragility fractures and respiratory distress). The survival of the affected children depends on the early total parathyroidectomy. Prenatal genetic test or genetic test at birth are recommended in case of both parents proved to be carriers of CaSR mutations and/or present clinical signs of FHH.

The key statements for differential diagnosis and indications for genetic testing of FHH and PHPT are presented in Table 2.

**Table 2:** Key statements for differential diagnosis and indications for genetic testing of FHH and PHPT

• The hallmark of FHH is a low (<0.01) CaCrCR, but ≈20% of FHH patients have a CaCrCR > 0.01 [55].
• A replete vitamin D status is required for the accurate establishment of CaCrCR and the diagnosis of both FHH and PHPT.
• Renal impairment and thiazide use may result in false CaCrCR.
• The testing criteria for genetic diagnosis of FHH is CaCrCR < 0.02 [55].
• The recommended sequence of genetic testing follows the natural prevalence of various FHH subtypes, that is (1) *CASR* for FHH1, (2*) AP2S1* for FHH3 and (3) *GNA11* for FHH2.
• The presence of a parathyroid adenoma does not rule out the diagnosis of FHH.

*FHH* familial hypocalciuric hypercalcemia, *PHPT* primary hyperparathyroidism, *CaCrCR* calcium-to-creatinine clearance ratio, *CaSR* calcium-sensing receptor

## *CaSR* gene polymorphisms—clinical consequences

Several CaSR polymorphisms have been identified in the intracellular domain of the *CaSR* gene. Among them, three clustered polymorphisms have been widely investigated in the past few decades (A986S (rs1801725), R990G (rs1042636) and Q1011E (rs1801726)). The world-wide average allele frequencies of these genetic variants were reported as 0.1259 for A986S, 0.1456 for R990G and 0.9462 for Q1011E (https://gnomad.broadinstitute.org/).

These polymorphisms modify the three-dimensional structure of cytoplasmic tail of CaSR; therefore, it was presumed that they may influence the intracellular CaSR signalling. However, the data are conflicting regarding the functional consequences of these structural modifications. On the basis of in vitro functional characterisation of these genetic variants, it became widely accepted that they have no significant effect on CaSR function [40]. Furthermore, we and others have provided evidence that the effects of the *CaSR* gene mutations are not influenced by the presence of these variants [41, 42].

However, in the last few years, polymorphisms of the CaSR became the target of numerous oncological investigations as they may contribute to the development of cancer-induced hypercalcemia and consequently influence the clinical outcome of different malignant diseases. The independent effect of hypercalcemia on cancer development and prognosis is widely investigated in colorectal-, and breast cancers; however, it still remained undetermined whether the functional status of CaSR could be related to cancer outcomes in affected patients. Among the three above-clustered CaSR polymorphisms, A986S (rs1801725) and Q1011E (rs1801726) have been previously associated with higher serum calcium level [43, 44]. On the contrary, R990G polymorphism seems to produce gain-of CaSR function and consequently associated with hypercalciuria in normal population and increased risk of renal stones in patients with PHPT [45, 46].

According to a recent meta-analysis, Q1011E (rs1801726) polymorphism has been associated with higher colorectal cancer risk in the distal colon site (OR: 1.418, 95% C.I.: 1.017–1.977) while the presence of R990G (rs1042636) allele decreased the colorectal cancer risk both in proximal and distal colon. The most frequent polymorphism A986S (rs1801725) seems to have no effect on colorectal cancer risk in Caucasian population [47].

Elevated serum calcium has been coupled with poorer prognosis in breast cancer even without bone metastases, therefore polymorphisms previously predicted to decrease the sensitivity of the CaSR (A986S and Q1011E) are also in the focus in some oncological investigations concerning breast cancer. According to a recent paper, hypercalcemia in about 20% of breast cancer patients without bone metastases is partly due to the presence of A986S polymorphism [48].

## Medical treatment with allosteric modulators of the CaSR

Detailed knowledge about the structure and function of the CaSR promoted the development of compounds acting as allosteric modulators on the CaSR. Calcimimetic drugs have positive allosteric effects on the receptor ligand binding capacity, increasing the sensitivity of the CaSR to extracellular Ca^2+^ [49]. The prototype of this class of drugs is cinacalcet, which binds to the transmembrane domain of the CaSR. Its use is approved in patients with SHPT due to chronic end-stage kidney failure and in patients with PHPT for whom parathyroidectomy would be indicated on the bases of serum calcium levels, but in whom parathyroidectomy is clinically inappropriate or is contraindicated [50]. Concordant evidence suggests that calcimimetics also improve the clinical signs of symptomatic hypercalcemia in FHH patients. The recently approved etelcalcetide should be administered intravenously for the management of SHPT in adults on chronic haemodialysis. The binding site of etelcalcetide differs from that of cinacalcet, since etelcalcetide binds to the Cys482 residue within the CaSR so-called venus flytrap module [51]. The net effect of calcimimetics is lowering PTH secretion and decreasing chief cell proliferation in the parathyroid glands [52]. According to some recent evidence, calcimimetics may also act as pharmacochaperones, since the enhancing effect on the trafficking of the mutant receptors to the cell surface has been proved [53].

In contrast, calcilytic molecules have negative allosteric effects on CaSR. These drugs were originally developed for the treatment of osteoporosis, taking advantage of the anabolic effect of elevated PTH concentration. However, animal models did not confirm this hypothesis, therefore calcilytics remain possible drug candidates for treating ADH patients in whom calcium and vitamin D supplementation is not sufficient for the correction of hypocalcemia or even worsens hypercalciuria leading to concurrent nephrocalcinosis, renal stone formation and renal impairment [54].

## Abbreviations

ADH: Autosomal dominant hypocalcemia
ADIS: Agonist-driven insertional signalling
AP2: Adaptor-related protein complex 2
AP2σ: Adaptor-related protein complex 2, sigma subunit
CaCrCR: Calcium-to-creatinine clearance ratio
CaSR: Calcium-sensing receptor
FHH: Familial hypocalciuric hypercalcemia
Gα_11_: G-protein subunit α_11_
NSHPT: Neonatal severe hyperparathyroidism
PHPT: Primary hyperparathyroidism
PTH: Parathyroid hormone
ROMK: Renal outer-medullary K+ channel
SHPT: Secondary hyperparathyroidism
